# Enzyme-Treated Caviar Prevents UVB Irradiation-Induced Skin Photoaging

**DOI:** 10.3390/md20110685

**Published:** 2022-10-30

**Authors:** Jeongjin Park, Dakyung Kim, Minhee Lee, Sangshin Han, Woojin Jun, Hyun Mook Jung, Yean Kyoung Koo, Gwi Hwan Na, Sang Hun Han, Jehee Han, Ok-Kyung Kim

**Affiliations:** 1 Division of Food and Nutrition and Human Ecology Research Institute, Chonnam National University, Gwangju 61186, Korea; 2 Department of Medical Nutrition, Kyung Hee University, Yongin 17104, Korea; 3 Cosmaxbio, Seongnam-si 13486, Korea; 4 Almas Caviar, Hwaseong-si 18553, Korea

**Keywords:** caviar, UVB, oxidative stress, skin photoaging

## Abstract

For this research article, we investigated the protective effects of enzyme-treated caviar powder extract (CV) in ultraviolet B (UVB)-irradiated hairless mice and keratinocytes by confirming moisturizing-related factors and elasticity-related factors. UVB irradiation induced wrinkle formation, dehydration, oxidative stress, and inflammation in the dorsal skin of mice; however, these were suppressed in the CV-supplemented groups in UVB-irradiated hairless mice. Furthermore, in UVB-irradiated keratinocytes, CV treatment increased the antioxidant enzyme activities and the levels of sphingomyelin and hyaluronic acid and decreased the production of pro-inflammatory cytokines and the expression of IkB-α and p65 phosphorylation. These findings indicate that CV can directly protect keratinocytes against UVB irradiation-induced oxidative stress and inflammation. Therefore, we suggest that CV can protect against UVB-induced skin photoaging. Therefore, we suggest that caviar is effective for skin health by preventing UVB-induced skin photoaging.

## 1. Introduction

The skin is an important tissue that protects the body’s internal structures from external environmental factors, such as bacteria, chemicals, temperature, humidity, and ultraviolet (UV) radiation; however, it is susceptible to damage due to direct exposure to these factors [1,2]. Among these environmental factors, exposure to UV radiation can cause serious damage to the skin, a complex biologic process known as photoaging. Maintaining skin hydration and elasticity is the key to healthy skin. By contrast, photoaging is characterized by the formation of wrinkles and the loss of skin hydration and elasticity, leading to skin aging. Ultraviolet A (UVA; 320−400 nm) and ultraviolet B (UVB; 280−320 nm) reach the earth’s surface, and UVB can be absorbed by the epidermis, playing a major role in skin damage [3,4,5].

Keratinocytes play an essential role in skin protection and repair in the outermost layer of the skin, the epidermis. UVB irradiation induces the destruction of the antioxidant defense system of keratinocytes by overproduction of reactive oxygen species (ROS). This condition can stimulate the inflammatory response and cell damage, leading to keratinocyte function impairment [6]. Hyaluronic acid synthesized by hyaluronic acid synthase (HAS) 1, HAS2, and HAS3 in keratinocytes keeps the skin moist, whereas UVB-irradiated keratinocytes fail to maintain the moisture retention capacity of the skin [7]. Moreover, inflammatory mediators secreted from the epidermis can affect the dermis, which is mainly composed of matrix components produced by fibroblasts, such as collagen, the protein responsible for the structure, elasticity, and firmness of the skin. Collagen is the most abundant matrix component in the dermis where matrix metalloproteinases (MMPs) induced by UV irradiation are expressed and degrade collagen. The inflammatory mediators stimulate the expression and activation of MMPs via the cellular MAPK signaling pathway, exacerbating collagen degradation and wrinkle formation [8,9,10].

Caviar (lightly salted fish roe products of certain fish species, especially members of the Acipenseridae family) is known worldwide as a luxurious food. It is a health-promoting food due to its nutritional value and high content of amino acids, fatty acids, and vitamins; however, few studies have evaluated its function and mechanism because of its expense [11,12,13]. In this study, we investigated whether enzyme-treated caviar powder extract (CV) could protect the skin against UVB irradiation in hairless mice through the confirmation of moisturizing- and elasticity-related factors in the skin. We also confirmed the effect of CV treatment on UVB-irradiated HaCaT cells to demonstrate whether CV directly affects keratinocytes. This study can scientifically demonstrate the physiological effects of caviar, enhancing its value.

## 2. Results

### 2.1. CV Protected Skin against UVB Irradiation-Induced Oxidative Stress in Hairless Mice

**Figure 1:**
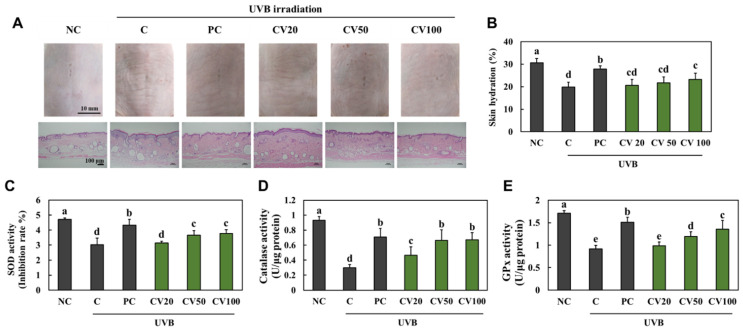
Effects of enzyme-treated caviar (CV) on morphological and histopathological changes (**A**), skin hydration (**B**), and antioxidant activities of SOD (**C**), CAT (**D**), and GPx (**E**) in the dorsal skin of UVB-irradiated hairless mice. Normal control (NC, −UVB), control (C, +UVB), L-ascorbic acid (positive control, PC; +UVB with dietary supplementation of L-ascorbic acid at 100 mg/kg/body weight [bw]), CV20 (+UVB with dietary supplementation of CV at 20 mg/kg/bw), CV50 (+UVB with dietary supplementation of CV at 50 mg/kg/bw), and CV100 (+UVB with dietary supplementation of CV at 100 mg/kg/bw). Values are presented as mean ± SD. Different letters (a > b > c > d > e) indicate a significant difference at *p* < 0.05 by Duncan’s multiple range test. SOD, superoxide dismutase; CAT, catalase; GPx, glutathione peroxidase.

The UVB-irradiated control group (C, +UVB) showed wrinkle formation, increased epidermal thickness (Figure 1A), and decreased skin hydration (Figure 1B) compared with the normal control group (NC, −UVB). In addition, the UVB-irradiated control group had decreased activities of antioxidant enzymes, including superoxide dismutase (SOD; Figure 1C), catalase (CAT; Figure 1D), and glutathione peroxidase (GPx; Figure 1E) compared with the normal control group. However, both the group given a dietary supplement of vitamin C at 100 mg/kg (positive control, PC) and the CV-supplemented groups showed attenuation of wrinkle formation and histopathological changes induced by UVB irradiation (Figure 1A). Moreover, the vitamin C-supplemented group and the groups that received CV (especially at 100 mg/kg/body weight [bw]) had increased skin hydration and activities of antioxidant enzymes compared with the UVB-irradiated control group (*p* < 0.05) (Figure 1B−E). These results suggest that CV protected skin against UVB irradiation-induced oxidative stress in hairless mice.

### 2.2. CV Suppressed UVB Irradiation-Induced Inflammation in Hairless Mice

**Figure 2:**
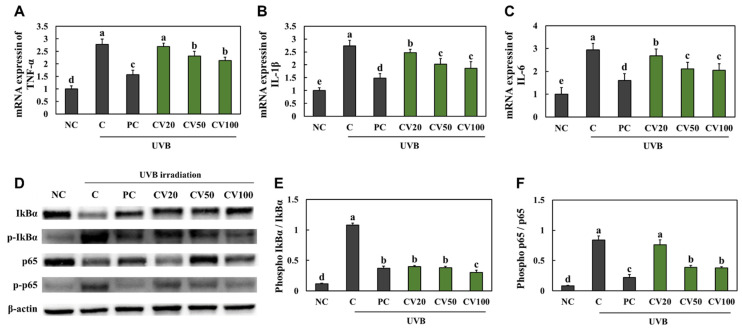
Effects of enzyme-treated caviar (CV) on mRNA expression of *TNF-α* (**A**), *IL-1β* (**B**), and *IL-6* (**C**) and expression of IkB-α and p65 phosphorylation ((**D**), band image; (**E**), quantification of p-IkB-α/total IkB-α; (**F**), quantification of p-p65/total p65) in the dorsal skin of UVB irradiation-induced photoaging in mice. Normal control (NC, −UVB), control (C, +UVB), L-ascorbic acid (positive control, PC; +UVB with dietary supplementation of L-ascorbic acid at 100 mg/kg/body weight [bw]), CV20 (+UVB with dietary supplementation of CV at 20 mg/kg/bw), CV50 (+UVB with dietary supplementation of CV at 50 mg/kg/bw), and CV100 (+UVB with dietary supplementation of CV at 100 mg/kg/bw). Values are presented as mean ± SD. Different letters (a > b > c > d > e) indicate a significant difference at *p* < 0.05 by Duncan’s multiple range test.

We investigated whether CV had anti-inflammatory effects on UVB irradiation-induced inflammation in hairless mice. We found that the mRNA expression levels of pro-inflammatory cytokines, *tumor necrosis factor-*α (*TNF-α*), *interleukin* (*IL*)*-1β*, and *IL-6*, were significantly increased in the UVB-irradiated control group compared with the normal control group (Figure 2A−C). Moreover, the expression of IkB-α and p65 phosphorylation was significantly increased in the UVB-irradiated control group compared with the normal control group (Figure 2D−F); however, the mRNA expression levels of pro-inflammatory cytokines and expression of IkB-α and p65 phosphorylation were significantly decreased in the vitamin C-supplemented group and the CV-supplemented groups compared with the UVB-irradiated control group (*p* < 0.05; Figure 2A−F). Thus, our findings indicated that CV supplementation inhibited inflammation in UVB-irradiated skin.

### 2.3. CV Increased the Activation of Elasticity Factors in UVB-Irradiated Hairless Mice

**Figure 3:**
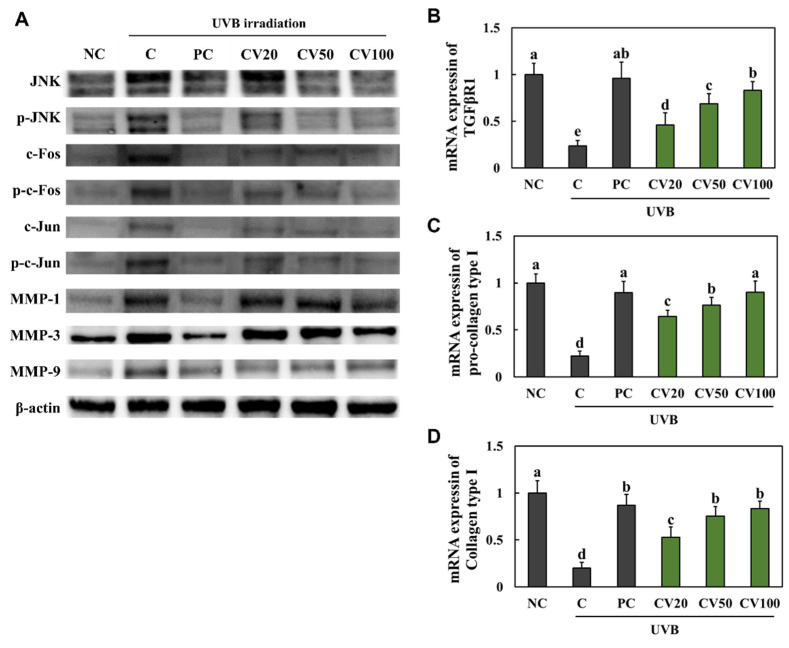
Effects of enzyme-treated caviar (CV) on protein expression of JNK/c-FOS/c-Jun/MMPs pathway (**A**) and mRNA expression of *TGF-β RI* (**B**), *pro-collagen type I* (**C**), and *collagen type I* (**D**) in the dorsal skin of UVB-irradiated hairless mice. Normal control (NC, −UVB), control (C, +UVB), L-ascorbic acid (positive control, PC; +UVB with dietary supplementation of L-ascorbic acid at 100 mg/kg/body weight [bw]), CV20 (+UVB with dietary supplementation of CV at 20 mg/kg/bw), CV50 (+UVB with dietary supplementation of CV at 50 mg/kg/bw), and CV100 (+UVB with dietary supplementation of CV at 100 mg/kg/bw). Values are presented as mean ± SD. Different letters (a > b > c > d > e) indicate a significant difference at *p* < 0.05 by Duncan’s multiple range test.

We measured the expression levels of proteins involved in the wrinkle formation pathway; c-Jun *n*-terminal kinase (JNK)/c-FOS/c-Jun/MMPs, and the mRNA expression levels of elasticity factors; transforming growth factor-β receptor I (*TGF-β RI*), *pro-collagen type I*, and *collagen type I* in the dorsal skin tissues. We found that the protein phosphorylation of JNK, c-FOS, and c-Jun and the protein expression of MMPs were increased in the UVB-irradiated control group compared with the normal control group (Figure 3A and Figure S1). The mRNA expression levels of *TGF-β RI, pro-collagen type I*, and *collagen type I* were decreased in the UVB-irradiated control group compared with the normal control group (Figure 3B−D). However, the protein phosphorylation of JNK, c-FOS, and c-Jun and the protein expression of MMPs were decreased in the vitamin C-supplemented group and the CV-supplemented groups compared with the UVB-irradiated control group (Figure 3A). Moreover, the mRNA expression levels of *TGF-β RI*, *pro-collagen type I*, and *collagen type I* were increased in the vitamin C-supplemented group and the CV-supplemented groups compared with the UVB-irradiated control group (Figure 3B−D).

### 2.4. CV Increased Moisturizing Capacity in UVB-Irradiated Hairless Mice

**Figure 4:**
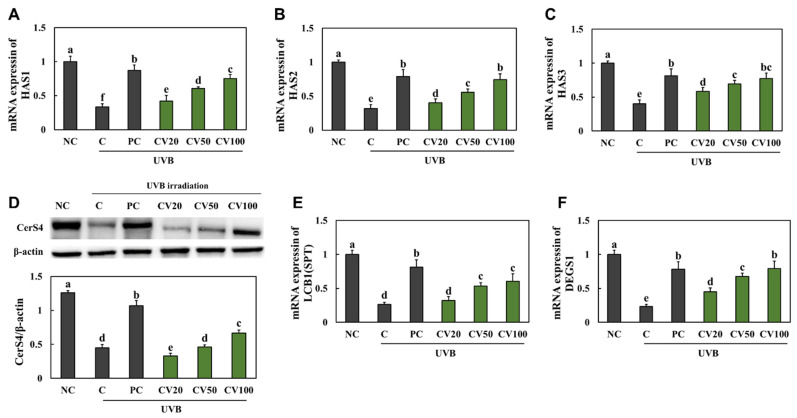
Effects of enzyme-treated caviar (CV) on mRNA expression of *HAS1* (**A**), *HAS2* (**B**), and *HAS3* (**C**), protein expression of CerS4 (**D**), and mRNA expression of *SLC35D1* (**E**) and *DEGS1* (**F**) in the dorsal skin of UVB-irradiated hairless mice. Normal control (NC, −UVB), control (C, +UVB), L-ascorbic acid (positive control, PC; +UVB with dietary supplementation of L-ascorbic acid at 100 mg/kg/body weight [bw]), CV20 (+UVB with dietary supplementation of CV at 20 mg/kg/bw), CV50 (+UVB with dietary supplementation of CV at 50 mg/kg/bw), and CV100 (+UVB with dietary supplementation of CV at 100 mg/kg/bw). Values are presented as mean ± SD. Different letters (a > b > c > d > e) indicate a significant difference at *p* < 0.05 by Duncan’s multiple range test.

We found that the mRNA expression levels of *HAS1* (Figure 4A), *HAS2* (Figure 4B), *HAS3* (Figure 4C), *SLC35D1* (Figure 4E)*,* and *DEGS1* (Figure 4F) and the protein expression level of ceramide synthase 4 (CerS4) (Figure 4D) were significantly decreased in the UVB-irradiated control group compared with the normal control group; however, they were increased in the vitamin C-supplemented group and the CV-supplemented groups compared with the UVB-irradiated control group (*p* < 0.05). These data indicate that CV supplementation can suppress the degradation of collagens in UVB-irradiated skin.

### 2.5. CV Suppressed UVB Irradiation-Induced Oxidative Stress and Inflammation in Keratinocytes

**Figure 5:**
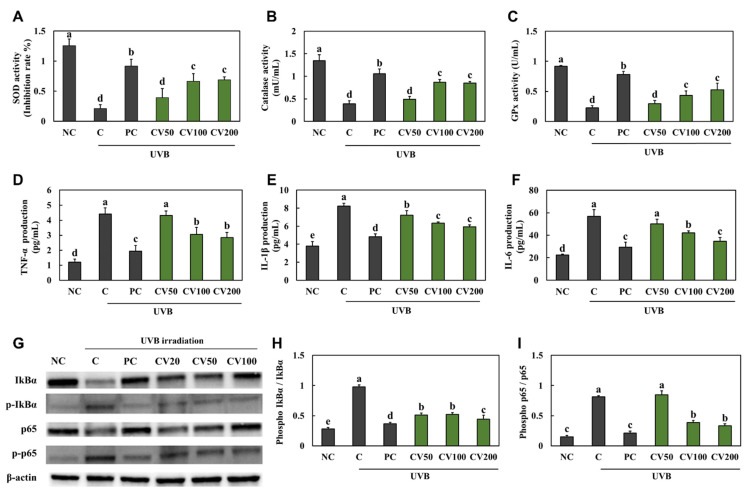
Effects of enzyme-treated caviar (CV) on antioxidant activities of SOD (**A**), CAT (**B**), and GPx (**C**), production of TNF-α (**D**), IL-1β (**E**), and IL-6 (**F**), and phosphorylation expression of IkB-α and p65 ((**G**), band image; (**H**), quantification of p-IkB-α/total IkB-α; (**I**), quantification of p-p65/total p65) in UVB-irradiated HaCaT cells. Normal control (NC, −UVB), control (C, +UVB), L-ascorbic acid (positive control, PC; +UVB with L-ascorbic acid treatment at 100 μg/mL), CV50 (+UVB with treatment of CV at 50 μg/mL), CV100 (+UVB with treatment of CV at 100 μg/mL), and CV200 (+UVB with treatment of CV at 200 μg/mL). Values are presented as mean ± SD. Different letters (a > b > c > d > e) indicate a significant difference at *p* < 0.05 by Duncan’s multiple range test.

CV treatment showed no signs of cytotoxicity up to 400 μg/mL in HaCaT cells (Figure S2), we thus investigated the effect of 50, 100, and 200 μg/mL CV in HaCaT cells. In the UVB-irradiated HaCaT cells (C, +UVB), the activities of antioxidant enzymes, including SOD (Figure 5A), CAT (Figure 5B), and GPx (Figure 5C), were decreased compared with the normal HaCaT cells (NC, −UVB). The production of pro-inflammatory cytokines, TNF-α, IL-1β, and IL-6, were significantly increased in the UVB-irradiated HaCaT cells compared with the normal HaCaT cells (Figure 5D−F). Moreover, the expression of IkB-α and p65 phosphorylation was significantly increased in the UVB-irradiated HaCaT cells compared with the normal HaCaT cells (Figure 5G−I). However, vitamin C- or CV-treated cells showed an increase in the activities of antioxidant enzymes (Figure 5A−C) and a decrease in the production of pro-inflammatory cytokines and expression of IkB-α and p65 phosphorylation (Figure 5D−F) (*p* < 0.05). These results suggest that CV can protect keratinocytes against UVB irradiation-induced oxidative stress and inflammation.

### 2.6. CV Increased the Moisturizing Capacity in UVB-Irradiated Keratinocytes

**Figure 6:**
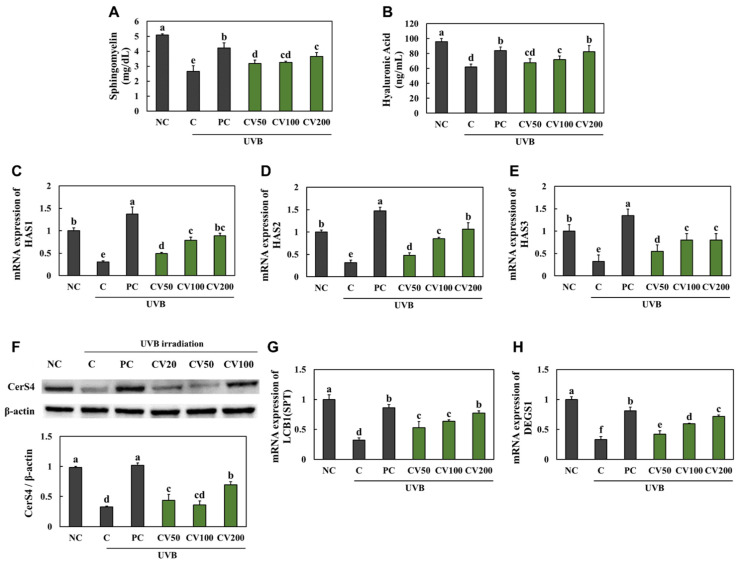
Effects of enzyme-treated caviar (CV) on levels of sphingomyelin (**A**) and hyaluronic acid (**B**), mRNA expression of *HAS1* (**C**), *HAS2* (**D**), and *HAS3* (**E**), protein expression of CerS4 (**F**), and mRNA expression of *SLC35D1* (**G**) and *DEGS1* (**H**) in UVB-irradiated HaCaT cells. Normal control (NC, −UVB), control (C, +UVB), L-ascorbic acid (positive control, PC; +UVB with L-ascorbic acid treatment at 100 μg/mL), CV50 (+UVB with treatment of CV at 50 μg/mL), CV100 (+UVB with treatment of CV at 100 μg/mL), and CV200 (+UVB with treatment of CV at 200 μg/mL). Values are presented as mean ± SD. Different letters (a > b > c > d > e) indicate a significant difference at *p* < 0.05 by Duncan’s multiple range test.

We investigated whether CV had a direct effect on the hydration of keratinocytes. The levels of sphingomyelin (Figure 6A) and hyaluronic acid (Figure 6B), mRNA expression levels of HAS1 (Figure 6C), HAS2 (Figure 6D), HAS3 (Figure 6E), SLC35D1 (Figure 6G), and DEGS1 (Figure 6H), and protein expression level of CerS4 (Figure 6F) were significantly decreased in the UVB-irradiated HaCaT cells compared with the normal HaCaT cells. However, they were significantly increased in the vitamin C- or CV-treated cells compared with the UVB-irradiated cells (Figure 6) (*p* < 0.05).

## 3. Discussion

Assays performed by Lee et al. [13] demonstrated that caviar extracted with 70% ethanol inhibited UVB-induced skin aging by stimulation of adipocyte differentiation and adiponectin production. To identify caviar’s components inhibiting skin aging, we demonstrated the skin protective effect of CV, which excluded the lipid components after supercritical CO_2_ extraction. We found that the protein amount was increased by supercritical CO_2_ extraction than by 70% ethanol extraction (Table S1) enzyme-treated extraction had free amino acids, especially high leucine (Table S2). Murakami et al. [14] demonstrated that oral administration of combinations of branched-chain amino acid, and glutamine or proline improved skin collagen protein synthesis, but a single oral administration of the amino acid had no effect. Therefore, we expected that the enzyme-treated extraction contains various free amino acids to improve UVB irradiation-induced skin photoaging.

It is well known that UVB irradiation induces ROS overproduction, inducing skin damage. When ROS production levels exceed a certain threshold, the overproduction of ROS interferes with the cell’s antioxidant defenses, exacerbating the decline in the activity of antioxidant enzymes, a phenomenon known as oxidative stress [15]. Filip et al. [16] found that single-dose 240 mJ/cm^2^ UVB irradiation in the dorsal skin of mice resulted in an increase in GPx activity compared with normal control mice. However, UVB irradiation for 5 days (Ahn et al. [17]) and UVB irradiation for 10 weeks (Kong et al. [18]) induced a decrease in GPx activity in the dorsal skin. Therefore, the effect on the antioxidant system depends on the frequency and duration of UVB irradiation. In the present study, we showed that UVB irradiation for 8 weeks caused inhibition of antioxidant enzyme activities in the dorsal skin of hairless mice. However, consistent with our expectations, dietary supplementation of CV protected against UVB irradiation-induced oxidative stress in the dorsal skin. Katayama et al. [19] demonstrated the antioxidative effects of amino acids on hydrogen peroxide (H_2_O_2_)-induced oxidative stress in human intestinal epithelial cells. Therefore, we suggest that CV suppresses oxidative stress caused by UVB irradiation because CV contains free amino acids.

There is a close relationship between oxidative stress and inflammation in the pathogenesis of various skin diseases [20]. UVB-mediated oxidative stress stimulates a number of transcription factors, including active protein 1 (AP-1) and nuclear factor kappa B (NF-κB) p65, involved in the inflammatory response by ROS accumulation [5,9,20]. We found that the mRNA expression of pro-inflammatory cytokines and expression of IkB-α and p65 phosphorylation in UVB-irradiated dorsal skin and the production of pro-inflammatory cytokines and expression of IkB-α and p65 phosphorylation in UVB-irradiated keratinocytes were increased, indicating that UVB irradiation caused inflammation in the epidermis. Moreover, it was confirmed that the production of sphingomyelin and hyaluronic acid, which contribute to skin moisturizing, decreased in UVB-irradiated keratinocytes, suggesting the impairment of keratinocyte function by UVB irradiation. However, our data showed that CV suppressed the expression of inflammatory factors in both the UVB-irradiated dorsal skin of mice and UVB-irradiated keratinocytes and increased the production of sphingomyelin and hyaluronic acid in UVB-irradiated keratinocytes. These findings indicate that CV attenuated inflammation and cell damage by directly inhibiting oxidative stress in keratinocytes.

A typical symptom of photoaging is the formation of deep wrinkles. The pro-inflammatory cytokines secreted from keratinocytes can stimulate fibroblasts in the dermis and activate MMPs, including MMP-1 (collagenase), MMP-3 (stromelysin-1), and MMP-9 (gelatinase), by JNK-mediated-c-Fos and c-Jun. These upregulated MMPs degrade matrix components, especially collagen, resulting in wrinkle formation [21,22,23]. Our data showed that dietary supplementation of CV suppressed wrinkle formation, phosphorylation of JNK, c-Fos, and c-Jun, and the expression of MMPs in the UVB-irradiated dorsal skin of mice. We suggest that CV treatment can suppress wrinkle formation by directly inhibiting the development of oxidative stress and inflammation in keratinocytes.

## 4. Materials and Methods

### 4.1. Preparation of CV

The caviars were obtained from a sturgeon farming company (Almascaviar, Inc., Seoul, Korea). Caviar was freeze-dried and used for the preparation. The dried caviar (5 kg) was extracted using a supercritical CO_2_ extraction system (Ilsin Autoclave, Inc., Daejeon, Korea). The extraction conditions were a pressure of 400 bar, a temperature of 40 °C, a flow rate of 60 mL/min, and an extraction time of 300 min to separate residue and lipids. The residue, excluding the lipid components, was extracted with enzymes in 10 volumes (*w/v*) of distilled water at pH 7, 50 °C for 4 h, followed by heat-induced inactivation of the enzymes at 100 °C for 1 h. The enzymes used were Prozyme1000L (Vision Biochem Co., Ltd., Seoul, Korea) and Protamex (Novozymes Co., Ltd., Bagsvaerd, Denmark). The enzyme-treated product was filtered and concentrated and then lyophilized to obtain enzyme-treated powder (1 kg).

### 4.2. Animals and UVB Irradiation

The Institutional Animal Care and Use Committee of Kyung Hee University approved the protocol (KHGASP-21–325) for the animal studies. The animals were cared for in accordance with the “Guidelines for Animal Experiments” established by the university. Male hairless mice (SKH-1) (5-week-old) were purchased from SaeRon Bio (Uiwang, Korea) and housed in cages under automatically controlled temperature (23 ± 2 °C), humidity (50 ± 5%), and lighting (12:12-h light−dark cycle). All animals were acclimatized for 7 days before the experiment, fed standard pellet chow, and given fresh water ad libitum. All mice were randomly divided into six groups (eight animals/group): normal control (−UVB), control (+UVB), L-ascorbic acid (positive control, PC; +UVB with dietary supplementation of L-ascorbic acid at 100 mg/kg/body weight [bw]), CV20 (+UVB with dietary supplementation of CV at 20 mg/kg/bw), CV50 (+UVB with dietary supplementation of CV at 50 mg/kg/bw), and CV100 (+UVB with dietary supplementation of CV at 100 mg/kg/bw).

Skin wrinkles were induced using a UVB lamp (Sankyo Denki Co., Yokohama, Japan) according to methods described previously [24]. The minimal erythematous dose (MED) was set at 150 mJ/cm^2^. Each time intensity of UVB irradiation was 1 MED (150 mJ/cm^2^) at week 1; 2 MED (300 mJ/cm^2^) at week 2; 3 MED (450 mJ/cm^2^) at week 3; and 4 MED (600 mJ/cm^2^) at week 4 to 8. At the end of the 8 weeks, all animals were sacrificed, and the dorsal skin and blood (by orbital venipuncture) were collected for analysis.

### 4.3. Histological Observation

Dorsal skin tissues were fixed in 10% buffered formalin and then embedded in paraffin. The paraffin blocks were sliced into 4 μm sections that were subsequently stained with hematoxylin and eosin (H&E) for histopathological observation.

### 4.4. Measurement of Skin Hydration

Hydration of the dorsal skin surface was measured with the Howskin device (Seoul, Korea) according to previously described methods [24], under standardized conditions of 22–24 °C and 55–60% humidity.

### 4.5. Measurement of Antioxidant Enzyme Activity

Dorsal skin tissues were collected from mice, and the activities of SOD, CAT, and GPx were measured using respective assay kits purchased from BioVision, Inc. (Milpitas, CA, USA); superoxide dismutase 1 (SOD1) (Mouse) ELISA Kit, Catalase Activity Colorimetric/Fluorometric Assay Kit, and Glutathione Peroxidase Activity Colorimetric Assay Kit, according to methods described previously [24].

### 4.6. Cell Culture and Treatments

HaCaT cells were obtained from the American Type Culture Collection (ATCC, Manassas, VA, USA). The cells were cultured in Dulbecco’s minimal essential medium (Hyclone Laboratories, Logan, UT, USA) with 10% fetal bovine serum (Hyclone Laboratories, Logan, UT, USA), 100 mg/L penicillin–streptomycin (Hyclone Laboratories, Logan, UT, USA), and 2 mmol/L glutamine (Hyclone Laboratories, Logan, UT, USA). HaCaT cells were exposed to UVB (50 mJ/cm^2^) using a UVB, followed by treatment with 100 µg/mL L-ascorbic acid or CV. The treated HaCaT cells were then harvested for further assays.

### 4.7. Total RNA Isolation and Real-Time PCR

Total RNA was extracted from dorsal skin tissues and HaCaT cells and analyzed for expression of the genes TNF-α, IL-1β, IL-6, TGF-β RI, pro-collagen type I, collagen type I, HAS1, HAS2, HAS3, SLC35D1, DEGS1, and GAPDH, by real-time PCR according to methods described previously [24].

### 4.8. Protein Extraction and Western Blot Analysis

Proteins were extracted from dorsal skin tissues and HaCaT cells and analyzed for the expression of IkB-α, phospho-IkB-α, p65, phospho-p65, JNK, phospho-JNK, c-FOS, phospho-c-FOS, c-Jun, phospho-c-Jun, MMP-1, MMP-3, MMP-9, CerS4, and β-actin, according to methods described previously [24].

### 4.9. Measurement of Pro-Inflammatory Cytokines

The levels of TNF-α, IL-1β, and IL-6 in the HaCaT cells supernatant were measured using the DuoSet ELISA Kit (R&D Systems, Minneapolis, MN, USA) according to the manufacturer’s method.

### 4.10. Measurement of Sphingomyelin and Hyaluronic Acid

HaCaT cells were lysed, and the levels of sphingomyelin and hyaluronic acid were then determined using the Sphingomyelin Assay Kit (Abcam, Cambridge, UK) and the Hyaluronic Acid ELISA Kit (BioVision, Inc., Milpitas, CA, USA), respectively, according to the manufacturer protocols.

### 4.11. Statistical Analysis

All data are presented as mean ± standard deviation (SD). The data were statistically evaluated by Duncan’s multiple range test after one-way ANOVA using SPSS statistical procedures (SPSS PASW Statistic 23.0, SPSS, Inc., Chicago, IL, USA). Statistically significant differences were considered at *p* < 0.05.

## 5. Conclusions

In the present study, we investigated the protective effect of CV against UVB irradiation-induced skin photoaging. We demonstrated that CV supplementation suppressed UVB irradiation-induced wrinkle formation, oxidative stress, inflammation, and moisture loss. In UVB-irradiated keratinocytes treated with CV, the CV treatment reduced oxidative stress and inflammation, besides increasing the expression of moisturizing-related factors. These findings indicated that CV treatment could suppress wrinkle formation by directly inhibiting oxidative stress and inflammation in keratinocytes. This study provides scientific evidence for the beneficial effects of caviar on skin health.

## Data Availability

Not applicable.

## Supplementary Materials

The following supporting information can be downloaded at: https://www.mdpi.com/article/10.3390/md20110685/s1.
