# Supplementary material for: Enzyme-Treated Caviar Prevents UVB Irradiation-Induced Skin Photoaging
Source: Mar Drugs. 2022 Oct 30;20(11):685. doi: 10.3390/md20110685 (PMC9696482; doi:10.3390/md20110685)
Supplement: Supplementary file 1 [file marinedrugs-20-00685-s001.zip › marinedrugs-1972755-supplementary.pdf]

Table S1. Nutrients composition of caviar extracts.

Component	70% ethanol extract from caviar	Supercritical CO_2 extract from caviar
Moisture	55.96%	1.14%
Protein	23.98%	68.31%
Lipid	10.45%	9.10%
Ash	5.12%	12.28%
Carbohydrate	4.49%	9.17%

Table S2. Free amino acid contents of enzyme-treated caviar extracts.

Free amino acids	Lot1	Lot2	Lot3
Leu	13.37	10.91	14.10
Phe	5.39	4.35	5.72
Tyr	5.93	5.32	6.05
Ala	4.74	4.32	4.84
Ile	4.46	4.00	4.55
Val	3.16	2.72	0.88
Arg	4.12	3.19	4.34
Met	3.31	2.49	3.49
Lys	5.14	3.92	5.38
Ser	2.75	2.60	2.77
Thr	2.01	1.74	2.09
Asn	1.44	1.18	1.48
Gln	1.93	1.57	1.99
Glu	2.83	2.63	2.87
His	1.34	1.19	1.32
Asp	0.95	0.91	0.95
Trp	0.60	0.53	0.62
Gly	0.71	0.70	0.70
Pro	0.70	0.58	0.77
Cys	0.05	0.07	0.06

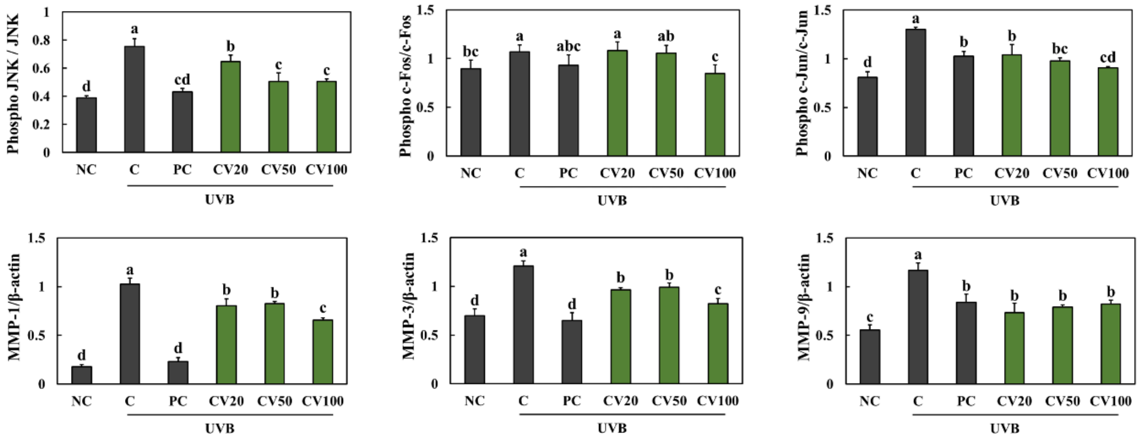

Figure S1. Quantification of the western blot bands relative expression in Figure 3A.

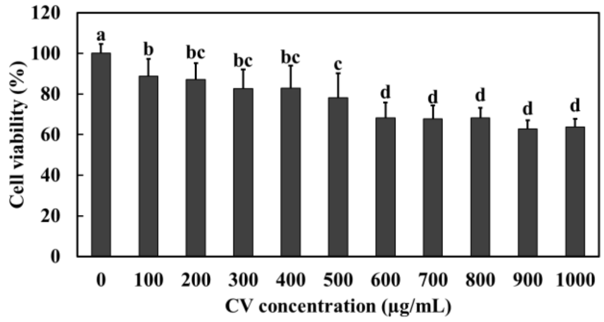

Figure S2. Effect of CV treatment in cell viability on HaCaT cells. Values are presented as mean ± SD. Different letters (a > b > c > d) indicate a significant difference at $p < 0.05$ by Duncan's multiple range test.
